# Large Encapsulated Apocrine Papilloma of the Breast Lacking Myoepithelial Markers: Case Report With Clinicopathologic and Immunohistochemical Studies

**DOI:** 10.1002/ccr3.9686

**Published:** 2024-12-05

**Authors:** Fatme Ghandour, Melissa Kyriakos Saad, Camil Chweiry, Elias Saikaly, Imad El Hajj

**Affiliations:** ^1^ Pathology Department, Saint George Hospital University Medical Center University of Balamand Beirut Lebanon; ^2^ General Surgery Department, Saint George Hospital University Medical Center University of Balamand Beirut Lebanon

**Keywords:** apocrine tumor, mastectomy, myoepithelial, papilloma of the breast

## Abstract

Encapsulated apocrine papilloma of the breast without myoepithelial cell markers is a rare occurrence. For its diagnosis, histopathological examination shows encapsulated apocrine papilloma lacking myoepithelial cell markers. To the best of our knowledge, this is the first documented case of an encapsulated apocrine papilloma entirely devoid of myoepithelial cell markers.

## 1 Introduction

Papillary lesions of the breast vary from benign papilloma to atypical papilloma or papilloma with ductal carcinoma in situ (DCIS) and papillary carcinoma. The common pattern in all lesions is the papillary architecture which is characterized by arborizing fronds having fibrovascular cores lined by epithelial and myoepithelial cells [1]. The papillary architecture is often evident, but the real challenge remains in identifying the myoepithelial cell layer in some cases, and the qualification and quantification of epithelial cell atypia in other cases [2]. In our case, a large encapsulated papillary lesion showed entire apocrine metaplasia, no structural complexity or atypia was noted, and myoepithelial cell markers were absent. The background breast tissue did not show atypical hyperplasia. To date, the negative myoepithelial immunohistochemical profile of benign papilloma remains a controversial topic. Herein, we present a case of an 85‐year‐old female patient presenting for enlarged left breast diagnosed with encapsulated apocrine papilloma of the breast lacking myoepithelial cell markers. The patient presented with progressively enlarging left breast and was found to have a cystic lesion in her left breast measuring 13 × 7 × 7 cm containing a 6 × 4 × 2.5 cm intracavitary papillary mass. Patient was managed surgically by a left modified radical mastectomy. To the best of our knowledge, this is the first case of an encapsulated purely apocrine papilloma with total lack of myoepithelial cell markers to be reported in the medical literature.

## 2 Case Report

**FIGURE 1:**
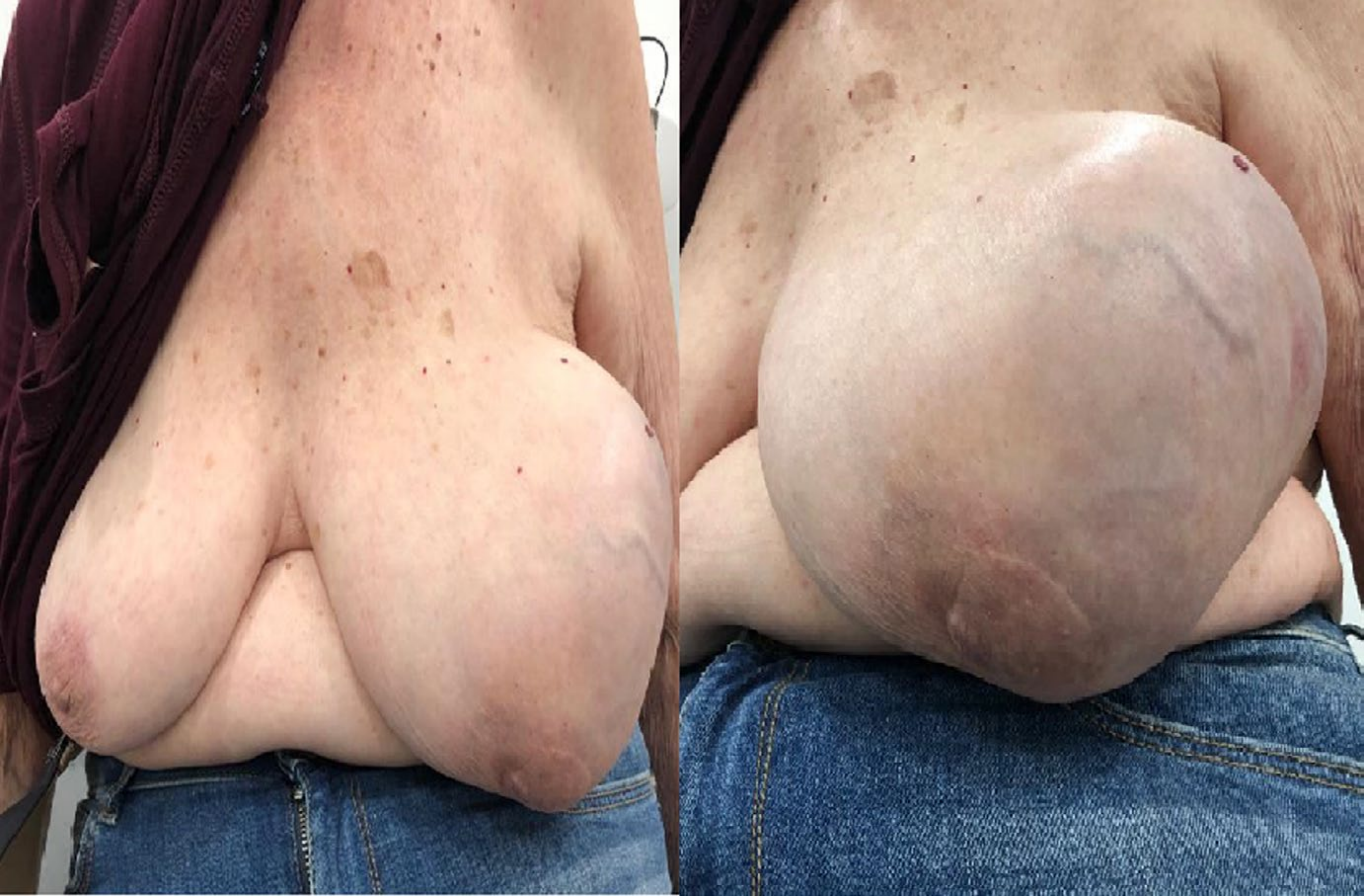
Diffusely enlarged breast with asymmetry on physical exam.

Case history and examination: 85‐year‐old female patient, gravida 4, para 4 known to have hypertension presenting for the management of progressively enlarging left breast. Patient denies a family history of breast cancer. She is a nonsmoker and has no history of oral contraceptive use. Patient reported breast feeding, no history of oral contraceptives. Physical examination revealed diffusely enlarged breast with asymmetry when compared to the contralateral breast (Figure 1), left breast large nonpainful palpable mass, soft in nature.

### 2.1 Methods

**FIGURE 2:**
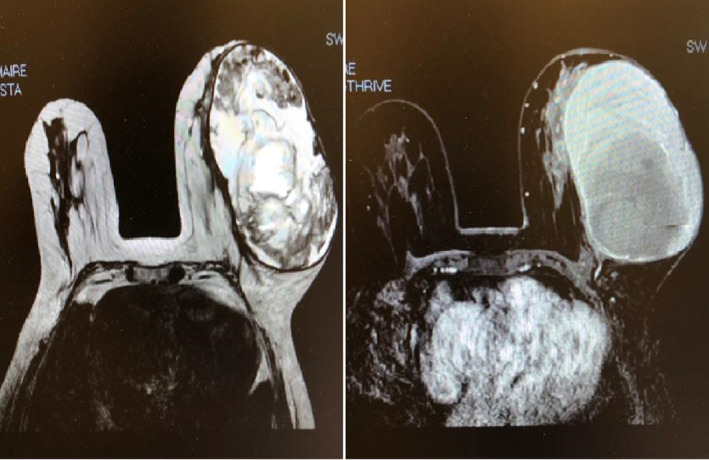
MRI breast showing huge cystic lesion in the left breast with septations within the cystic cavity and hyperdense material within this cystic cavity.

Radiologic investigations by breast MRI showed huge cystic lesion in the left breast with septations within the cystic cavity and hyperdense material within this cystic cavity (Figure 2). Chest abdomen pelvis computed tomography scan was negative for distant metastasis. Laboratory investigations were all within normal range, and the tumor markers including CA 15‐3, CA 27.29, CEA, CA 125, and CA 19‐9 were all within the normal range. The case was discussed within medical tumor board and decision was made for a left modified radical mastectomy.

### 2.2 Result

Patient underwent an uneventful left modified radical mastectomy.

### 2.3 Pathologic Findings

**FIGURE 3:**
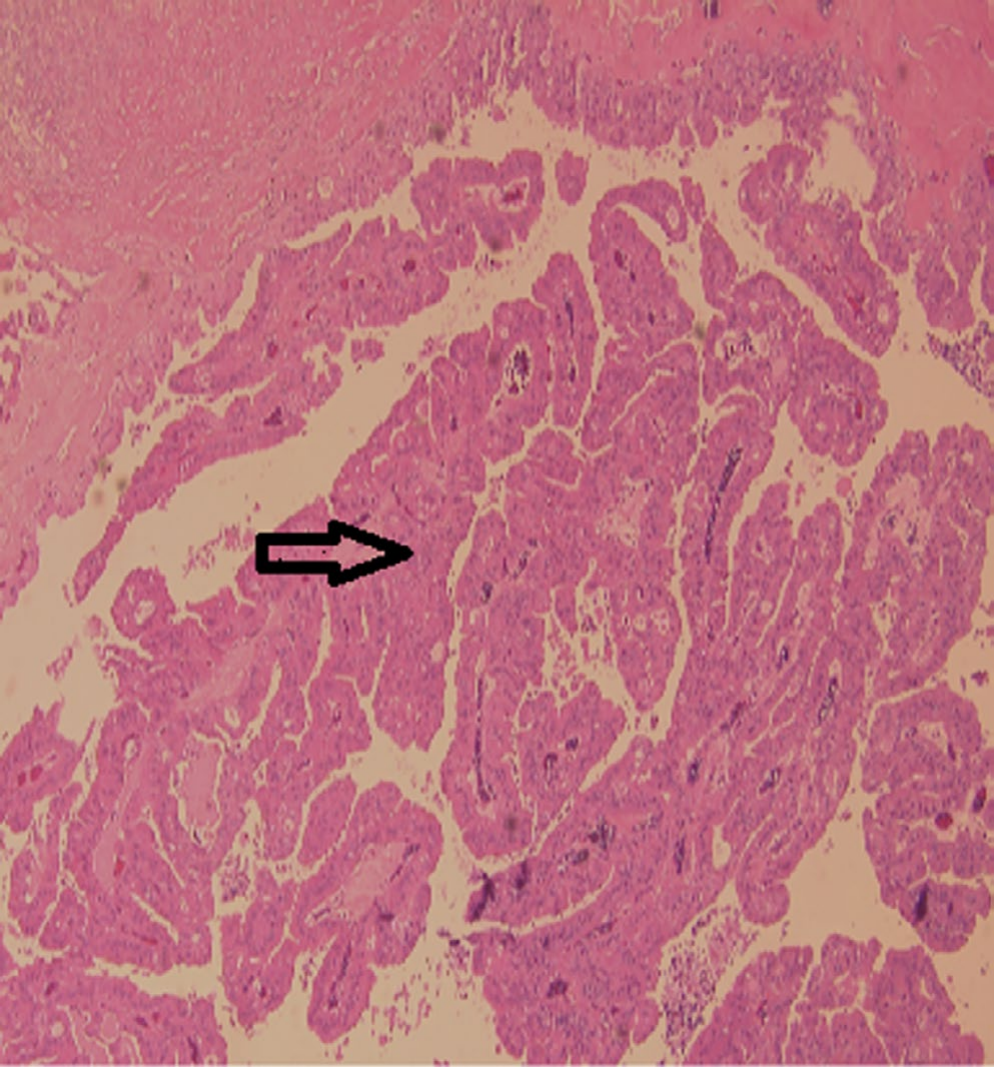
Prominent apocrine metaplasia arrow.

**FIGURE 4:**
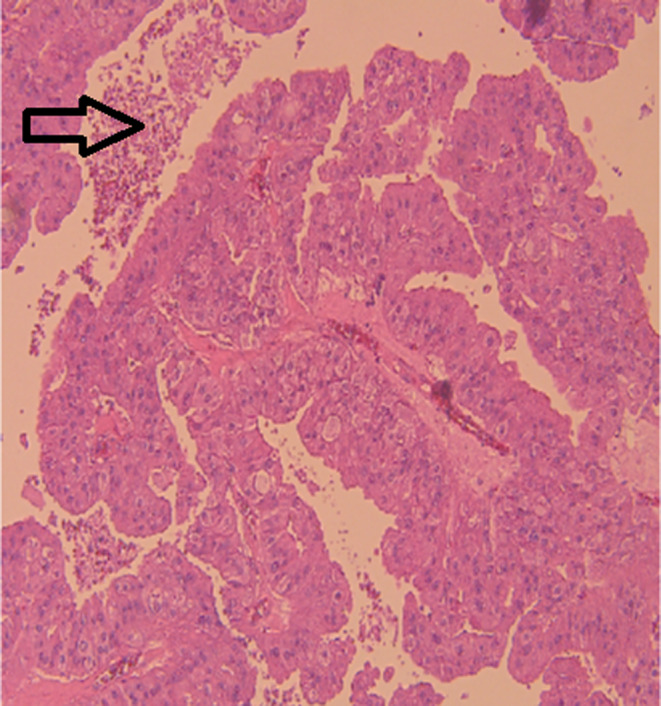
Abundant eosinophilic granular cytoplasm, absence of mitotic figures arrow.

**FIGURE 5:**
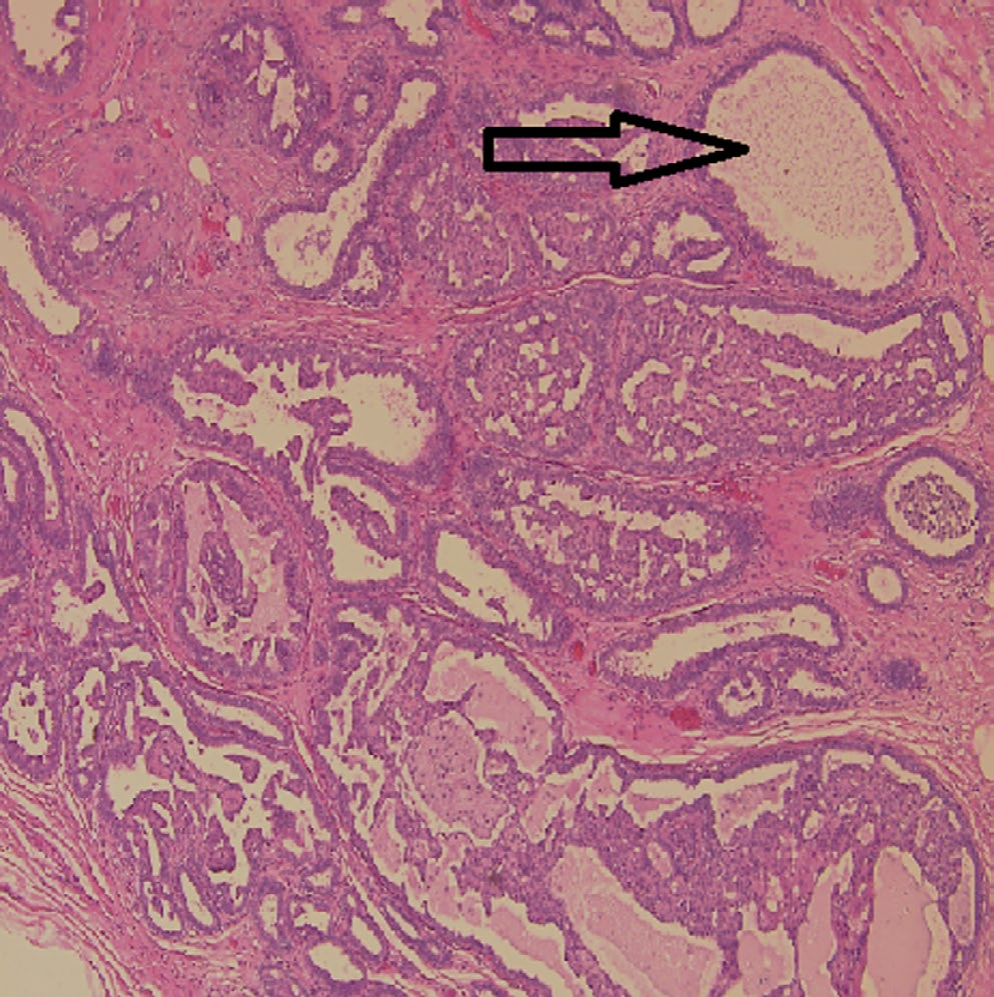
Dystrophic calcification, focal areas showing usual ductal hyperplasia arrow.

**FIGURE 6:**
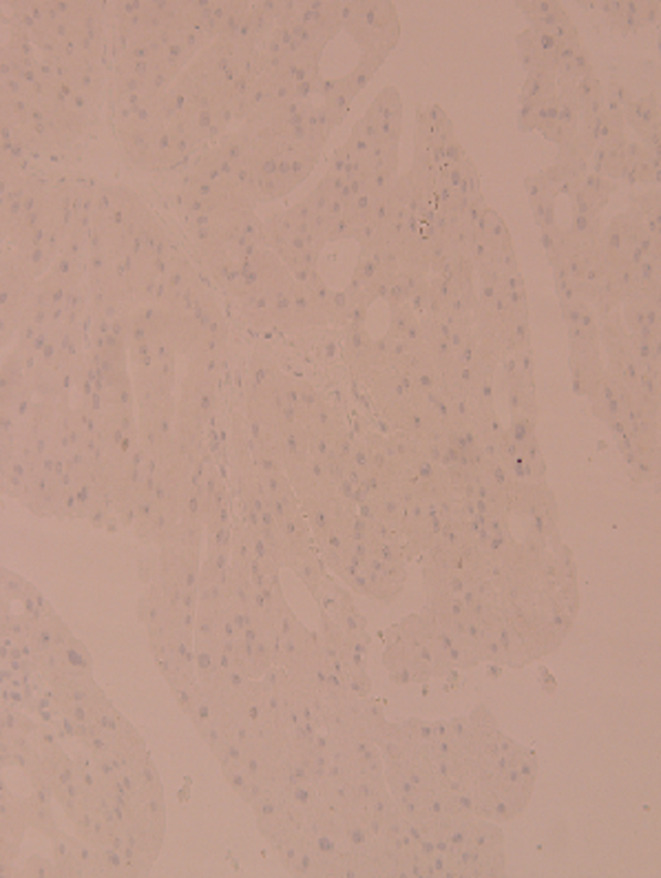
Negative results for p63.

**FIGURE 7:**
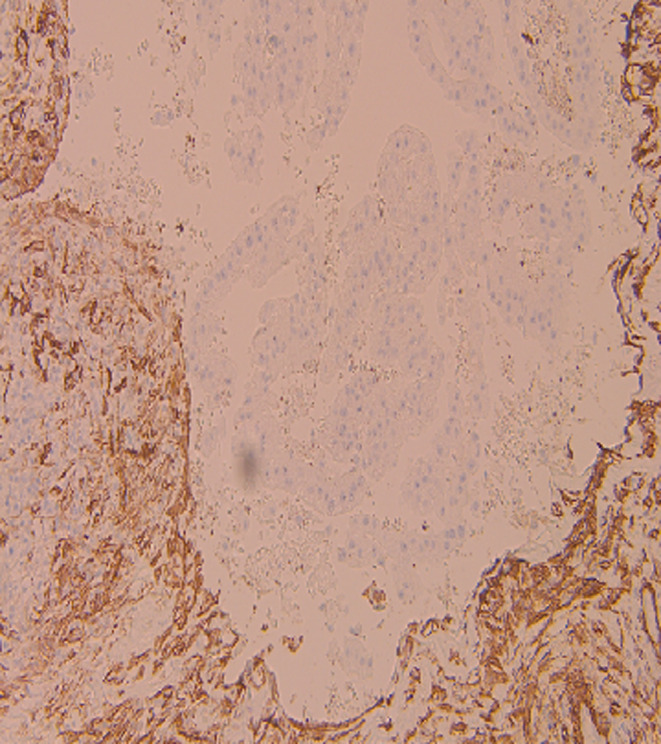
Negative for SMA.

**FIGURE 8:**
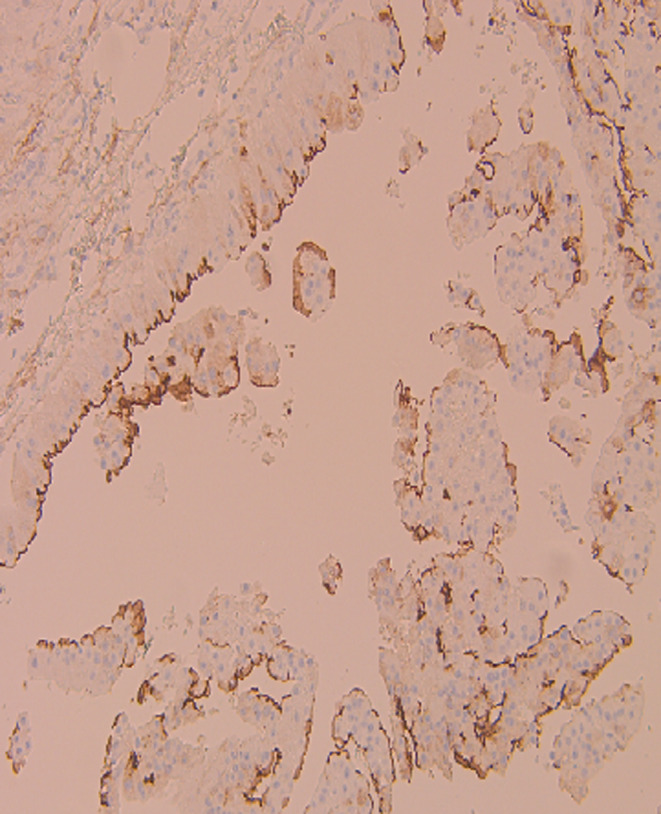
Negative for CD10.

The left breast measuring 19.5 × 14.5 × 9 cm covered by an ellipse of skin measuring 12 × 1 cm showed a 13 × 7 × 7 cm hemorrhagic cavity with a 6 × 4 × 2.5 cm friable papillary mass. On histopathology, a thick fibrous capsule focally lined by apocrine cells was noted which encased a papillary lesional growth with a prominent fibrovascular cores lined by epithelial cells showing prominent apocrine metaplasia but with minimal complexity (Figure 3). The apocrine cells were one cell layer thick. No tertiary branching or cribriform areas were seen. The cells exhibited uniform round nuclei, with pale chromatin, prominent nucleoli, abundant eosinophilic granular cytoplasm, low nucleo‐cytoplasmic ratio, and absence of mitotic figures (Figure 4). Some areas showed dystrophic calcification, and an area of ischemic infarction was seen. The background breast tissue was mostly fatty, with focal areas showing usual ductal hyperplasia. No atypical ductal proliferation or DCIS was seen (Figure 5). Immunohistochemical staining for myoepithelial cells was done and showed negative results for p63 (Figure 6), negative for SMA (Figure 7), and negative for CD10 (Figure 8). Also, the apocrine lining of the cyst wall was negative for the previously mentioned markers.

## 3 Discussion

The lack of myoepithelial cell markers in benign breast lesions should not be restricted to microglandular adenosis. It was actually first reported by Cserni in 2008 [3] who identified morphologically completely benign apocrine glands and cysts with no evidence of myoepithelium. He later reported two benign papillary apocrine lesions with no morphologic epithelial cell markers. One was negative for p63 and CD10, but was SMA, and S100 positive. The second showed focally positive myoepithelial cells using immunostains [4]. Cserni [4] suggested the idea that the lack myoepithelial markers could be due to significant distension or atrophy of cells resulting in myoepithelial cells being outside the plane of sectioning. Indeed, our case showed a large papillary lesion in a distended hemorrhagic cavity. Furthermore, Tramm, Kim, and Tavassoli [5] implied that an intricate interaction occurs between metaplastic apocrine cells and the surrounding myoepithelial environment, leading to possible change in the phenotypic myoepithelial cell expression.

Since encapsulated papillary lesions become problematic when glands are entrapped in an invasive pattern, our differential diagnosis included: papillary DCIS, encapsulated papillary carcinoma, and benign papilloma.

The fact that our case showed a well‐defined, thick fibrous capsule lined focally by epithelial cells lacking myoepithelial markers goes against papillary DCIS. In addition, no cytological atypia or cribriforimg was noted. Moreover, an encapsulated papillary carcinoma was less likely, because to date there are only few case reports showing entire apocrine metaplasia [6]. Also, no complexity of architectural growth pattern was noted on histologic examination, and the apocrine cells were morphologically bland, with no increase in nuclear to cytoplasmic ratio, bizarre looking nuclei, or abnormal mitosis. What also goes against encapsulated breast carcinoma was that the background breast tissue showed usual ductal hyperplasia with no areas of atypical hyperplasia or DCIS [6]. And even though myoepithelial cells were entirely absent, Cserni affirms that the partial presence of myoepithelial cells is not sufficient in ruling out malignancy [4].

## 4 Conclusion

To summarize, the diagnosis of papilloma should take into consideration the architectural pattern of growth, cellular morphology, and immunohistochemical profile. Even if myoepithelial cells are not present microscopically, a wide panel of immunostains could be helpful. The absence of myoepithelial cells should not be equated with malignancy. Absent myoepithelial cells in benign papillomas is a controversial topic that is becoming more evident as more cases are being reported in the medical literature.

Follow‐up of the patient and outcome: 1‐year follow‐up after surgery the patient has no complaints.

## Author Contributions

**Fatme Ghandour:** resources, writing – original draft, writing – review and editing. **Melissa Kyriakos Saad:** investigation, project administration, resources, writing – original draft, writing – review and editing. **Camil Chweiry:** resources, visualization, writing – original draft, writing – review and editing. **Elias Saikaly:** investigation, methodology, project administration, resources, supervision, visualization, writing – original draft, writing – review and editing. **Imad El Hajj:** methodology, supervision, validation, visualization, writing – review and editing.

## Consent

Written informed consent was obtained from the patient to publish this report in accordance with the journal's patient consent policy.

## Data Availability

The data that support the findings of this study are available on request from the corresponding author.
